# Comparison of Deep-Learning and Conventional Machine-Learning Methods for the Automatic Recognition of the Hepatocellular Carcinoma Areas from Ultrasound Images

**DOI:** 10.3390/s20113085

**Published:** 2020-05-29

**Authors:** Raluca Brehar, Delia-Alexandrina Mitrea, Flaviu Vancea, Tiberiu Marita, Sergiu Nedevschi, Monica Lupsor-Platon, Magda Rotaru, Radu Ioan Badea

**Affiliations:** 1 Computer Science Department, Technical University of Cluj-Napoca, 28 Memorandumului Street, 400114 Cluj Napoca, Romania; delia.mitrea@cs.utcluj.ro (D.-A.M.); flaviu.vancea@cs.utcluj.ro (F.V.); tiberiu.marita@cs.utcluj.ro (T.M.); sergiu.nedevschi@cs.utcluj.ro (S.N.); 2 Regional Institute of Gastroenterology and Hepatology, Iuliu Hatieganu University of Medicine and Pharmacy, Cluj-Napoca, 19-21 Croitorilor Street, 400162 Cluj-Napoca, Romania; monica.lupsor@umfcluj.ro (M.L.-P.); rbadea@umfcluj.ro (R.I.B.); 3 Medical Imaging Department, Iuliu Hatieganu University of Medicine and Pharmacy, Cluj-Napoca, 8 Babes Street, 400012 Cluj-Napoca, Romania; rotaru.magda@umfcluj.ro

**Keywords:** image processing, Convolutional Neural Networks (CNN), pattern recognition, ultrasound images, Hepatocellular Carcinoma (HCC), automatic diagnosis

## Abstract

The emergence of deep-learning methods in different computer vision tasks has proved to offer increased detection, recognition or segmentation accuracy when large annotated image datasets are available. In the case of medical image processing and computer-aided diagnosis within ultrasound images, where the amount of available annotated data is smaller, a natural question arises: are deep-learning methods better than conventional machine-learning methods? How do the conventional machine-learning methods behave in comparison with deep-learning methods on the same dataset? Based on the study of various deep-learning architectures, a lightweight multi-resolution Convolutional Neural Network (CNN) architecture is proposed. It is suitable for differentiating, within ultrasound images, between the Hepatocellular Carcinoma (HCC), respectively the cirrhotic parenchyma (PAR) on which HCC had evolved. The proposed deep-learning model is compared with other CNN architectures that have been adapted by transfer learning for the ultrasound binary classification task, but also with conventional machine-learning (ML) solutions trained on textural features. The achieved results show that the deep-learning approach overcomes classical machine-learning solutions, by providing a higher classification performance.

## 1. Introduction

One of the most severe diseases of liver is hepatic cirrhosis that changes the appearance and structure of the liver and blood vessels. Cirrhosis represents the basis from which HCC evolves, after a restructuring process, resulting in dysplastic nodules, which can transform into malignant tumors. HCC represents the most frequent malignant liver tumor, appearing in 75% of the liver cancer cases. It is one of the most common causes of death from liver diseases reported by WHO (World Health Organization) [1].

HCC is surrounded by cirrhotic liver tissue (parenchyma) that in some cases has a very similar visual aspect, making HCC areas hard to recognize by the human eye. An automatic process in which the doctor could select the region of interest, or in which regions of interest are generated automatically by a recognition module, together with a computer-aided diagnosis tool predicting the likelihood that an area belongs to HCC or not, would ease the medical practitioner’s work. The corresponding tool, based on computerized, non-invasive methods, can also replace the needle biopsy, the actual golden standard for HCC diagnosis, which is dangerous, as it could lead to the spread of the tumor inside the human body [1].

A common method of liver examination is ultrasonography. It is highly used because it is cheap, safe, non-invasive, and thus repeatable, suitable for patient disease monitoring. Other medical examination techniques, such as the Computer Tomography (CT), the Magnetic Resonance Imaging (MRI), the endoscopy or the Contrast Enhanced Ultrasonography (CEUS) are considered irradiating or expensive.

**Figure 1:**
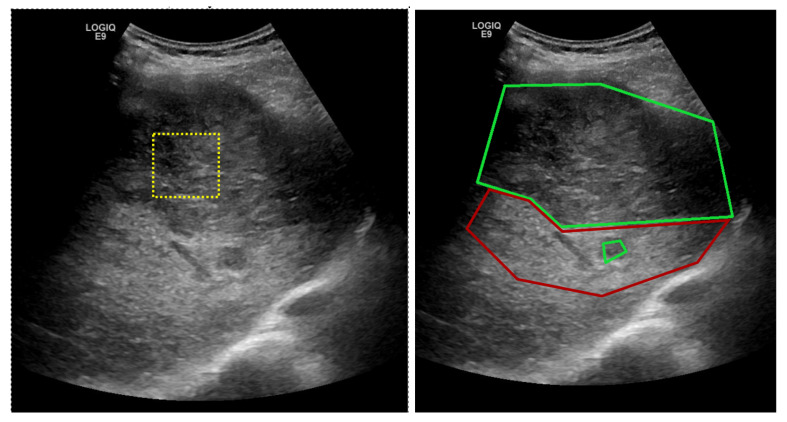
(**left**) Ultrasound image for which the medical specialist can select a region of interest for which the HCC/PAR confidence is needed; (**right**) Ground truth area—HCC area inside the large green polygon and PAR on which it has evolved delimited by the red polygon.

In ultrasound images, HCC usually appears as a hyperechogenic, inhomogeneous structure, due to the interleave of various tissue types, such as necrosis, fibrosis, active growth tissue, fatty cells [1]. As it can be noted from Figure 1, the visual aspect within ultrasound images of the PAR and HCC presents relevant features that characterize textures, such as finesse, coarseness, smoothness, surface granulation, randomness, irregularity.

The human-based analysis of the regions in the ultrasound images made in order to find problematic areas such as HCC or PAR can be aided by an automatic recognition method, such as the one proposed in this paper. The medical specialist could select regions of interest, as shown by the yellow patch in Figure 1 and obtain a probability score, displayed as a color confidence map, for each selected region. The proposed method classifies regions of interest from the ultrasound images and can provide a confidence map over the whole ultrasonographic image.

### 1.1. Deep-Learning Methods in Computer Vision Applications

Deep-learning methods are successfully used in computer vision tasks, such as object recognition, semantic segmentation of images, behavior recognition, generation of synthetic images [2,3], writer identity detection [4], face detection and identification [5], image classification [6], image segmentation [7], object detection [3]. For image classification, which is closely related to the subject of this paper, among the most popular networks we can mention VGGNet [8] that is a sequential network that contains blocks of 3 × 3 convolution layers in between a periodic max-pooling operation is done. The disadvantage of this network is that it has a high computational cost. GoogleNet [9] and its variants like InceptionNet-v3 [10] represent a milestone in the development of CNN architectures. The original GoogleNet contains 22 layers, so it is deeper than VGGNet, but it is also more computationally efficient. The achievement of a reduced computational cost is due to a good local network topology obtained by the usage of the so-called inception modules. As deeper neural networks are hard to train and may suffer from the problem of vanishing gradients that affects convergence, ResNet [11] includes a network topology that contains residual connections. These residual connections favor optimal training for really deep networks without degrading. ResNet models containing 18, 34, 50, 101 and even 152 layers have been proposed. Another approach that ensures the maximum information flow between the layers of the network is considered in DenseNet [12]. In this configuration, all layers that have equal feature-map sizes are directly connected with each other. To preserve the feed-forward nature of the network, each layer obtains additional inputs from all preceding layers and forwards its own feature maps to subsequent layers. A smaller CNN architecture, SqueezeNet [13] obtains a competitive accuracy with respect to large size CNN, while having fewer parameters and lower model size. The key design ideas considered by [13] are: the replacement of the 3 × 3 filters with 1 × 1 filters, the decrease in number of input channels to 3 × 3 filters and late downsample in the network so that convolution layers have large activation maps.

### 1.2. Deep-Learning Methods for Medical Image Analysis

The deep-learning techniques have been adopted in the field of medical image analysis in general [2,3,14] and for ultrasound images in particular [15], while conventional methods are also largely used [16]. A revision of deep-learning methods for radiological applications such as image classification, object detection or image segmentation in the X-ray images is provided by [2], while [3] presents how the application of deep-learning in computer vision has contributed to the development of assistive technologies with particular applications for object localization and recognition, scene understanding, human pose estimation and tracking, action and event recognition, anticipation.

A relevant and complex approach concerning the involvement of the deep-learning techniques in the characterization and recognition of the ultrasound images was presented in [17], where the authors implemented a deep CNN of type Inception-ResNet-v2, pre-trained using the ImageNet dataset. High-level features were computed based on sequences of liver B-mode ultrasound images. Then, a Support Vector Machines (SVM) classifier was trained on these high-level features, the purpose being to perform fatty liver recognition. A similar approach was presented in [18], to automatically diagnose cirrhosis from ultrasound images. Here, CNN networks were used for generating potentially important features from ultrasound images, then an SVM classifier was trained using these features, the resulting accuracy being 96%. Another method, based on deep-learning, which performs the detection of the cirrhosis severity grade for patients affected by chronic, B type hepatitis, within 2D shear wave elastographic images, was described in [19]. A CNN consisting of four convolutional layers and a single fully connected layer was adopted, the training set containing 1990 images belonging to 398 patients. The resulted performance, measured through the Area under the Receiver Operating Characteristic (AUC) metric, was always above 85% [19]. Another relevant approach was described in [20], where the authors aimed to detect tumor structures from breast ultrasound images, using a CNN-based technique, called Single Shot MultiBox Detector (SSD). The experimental dataset consisted of 579 benign and 464 malignant breast lesion cases. The proposed method provided better performance, in terms of precision and recall, than the other existing state of the art methods.

Regarding the role of the CNN-based techniques in the analysis and recognition of other types of medical images, an approach aiming to perform liver lesion patch-based recognition within CT images was described in [21], where the authors trained a CNN by using image patches, which were centered at each pixel. The patches contained both tumor as well as normal liver tissue. In [22] the authors performed liver tumor segmentation within CT images, with the aid of a 2D CNN. The corresponding algorithm defined a region of interest by employing a deformable registration of a baseline scan, manual tumor delineations, as well as the automatic segmentation of the liver. A voxel classifier was built with the aid of a CNN. In the experimental dataset, 67 tumors of 21 patients were included. Finally, the average overlap error of this method was assessed to 16.26%. An increased diagnostic performance concerning liver fibrosis staging was reported in [23], where the authors implemented a Deep Convolutional Neural Network (DCNN)-based technique. Gadoxetic, acid-enhanced, hepatobiliary phase magnetic resonance (MR) imaging was used in the experiments, the area under the Receiver Operating Characteristic(ROC) being 85%. The DCNN technique was also employed in [24], for detecting, within CT images, incipient pulmonary malignant nodules. The training dataset consisted of 62,492 regions of interest extracted from 40,772 nodules and 21,720 non-nodules, as part of the Lung Image Database Consortium (LIDC) data store. In this case, a maximum classification accuracy of 86.4% was achieved.

### 1.3. Classical Machine-Learning Methods for Medical Image Analysis

Texture-based methods in combination with traditional classification techniques have been widely employed, as well, for the purpose of automatic recognition of various affections, particularly of the tumor structures, within medical images [16,25,26,27]. Thus, in [25], the Wavelet transform, together with ANN were considered, to perform recognition of the liver tumors within ultrasound images. The parameters of the run-length matrix, as well as the Haralick features derived from the Gray Level Co-occurrence Matrix (GLCM) and also other gray level-based first order statistics were employed in combination with ANN classifiers, respectively Fisher Linear Discriminants, for performing automatic recognition of liver lesions based on ultrasound images in [26]. A more recent approach was described in [27], where the authors performed the recognition of the liver tumors by employing textural parameters computed from typical, as well as contrast enhanced CT images. The authors concatenated the textural features, which resulted from the original image, respectively from the contrast enhanced CT images acquired during the arterial and venous phases, obtaining a multi-phase feature vector. After feature selection, a C4.5 classifier was adopted, yielding an accuracy greater than 90%. The recently modified versions of the SVM classifier constitute a valuable alternative for high-performance recognition through traditional classifiers. In [28] the authors proposed a new formulation for the unconstrained convex minimization problem, in the case of the Lagrangian dual of the lately approached Twin Support Vector Machines (TWSVM). The newly defined technique was tested on multiple real-world datasets, including medical datasets [28] and provided a better performance than previously existing versions of the same classifier, such as classical SVM, TWSVM and Least-Squares TWSVM (LS-TWSVM).

However, lately, the deep-learning techniques, such as Recurrent Neural Networks (RNN), Stacked Denoising Autoencoders (SAE), Deep Belief Networks (DBN), as well as CNN were successfully implemented, in order to perform automatic diagnosis within medical images [29,30,31]. CNN have revealed, during the last decade, excellent results for image recognition, segmentation, detection, or feature extraction. Relevant research papers demonstrate the wide application of these networks within various types of medical images (such as X-ray, CT, MRI or histopathology images), for the diagnosis of different medical affections [17,21,22,23,24,29].

### 1.4. Contributions

Even if various approaches performing the recognition of the HCC malignant tumors, or of other significant liver affections, within ultrasound images, already exist [32,33], the deep-learning methods and their comparison with the traditional methods have not been yet sufficiently explored in this context. Taking into account the above described methods, one can conclude that no relevant approach exists in order to perform automatic HCC recognition within ultrasound images, by employing a systematic study upon the CNN architectures appropriate for this purpose, compared with the effect of the most significant texture/feature-based classification techniques.

Thus, the contributions of this paper are as follows:

- The proposal, development and experimentation of a multi-resolution CNN-based architecture suitable for highly textured ultrasound liver images. The proposed model combines parallel convolutions that capture multi-resolution features, residual connections that enable feature sharing between layers, and atrous convolutions for spatial pyramid pooling [7] that enlarge the field of view of the filters enabling a denser feature pool generation.
- In the context of a rich field of existing architectures used for image classification tasks, transfer learning procedure is employed on other five architectures: VGG [8], ResNet [11], Inception-V3 [10], SqueezeNet [13] and DenseNet [12]. A comparison of the classification performance in terms of accuracy, sensitivity, specificity and AUC is analyzed on the evaluation for the proposed method and the five fine-tuned architectures.
- The proposed deep-learning model is also compared with various conventional classification models that extract textural features and perform AdaBoost, SVM, Multi-layer Perceptron (MLP) or Random Forest (RF)-based classification. The explored textural features are (1) those derived from the GLCM matrix of order two and three [32], as well as from other statistical texture analysis methods applied on the original images; (2) the Shannon entropy computed after the recursive application of the Wavelet transform; (3) the Hurst fractal coefficient [34] and (4) Local Binary Patterns(LBP) [35].
- Two annotated ultrasound image datasets have been involved in this study. Due to medical and practical patient diagnosis reasons, the images have been collected with two different ultrasound devices (General Electric Logiq 9 and General Electric Logiq 7). The first dataset contains information collected from 200 patients, while the second employs 68 cases. For each patient at least 3 ultrasound images have been annotated by the medical specialists, so the HCC area within the image is well marked (as shown in Figure 1 right). Extensive experiments reveal the conclusion that deep-learning-based models overcome the classical machine-learning techniques bringing an improvement of 17% in sensitivity and an improvement of 20% in specificity.

## 2. Materials

The protocol for the communication and management of medical imaging is the Digital Imaging and Communications in Medicine (DICOM) [36]. For the ease of annotation and for further image analysis the DICOM images considered in this research were converted to BMP and then annotated by medical specialists using the VGG Image Annotator (VIA) tool [37,38]. The usage of two devices with different setups for the medical ultrasound analysis procedure has generated two datasets (we name them dataset GE7 and dataset GE9) on which the proposed method was tested. All the considered patients were biopsied for diagnostic confirmation.

### 2.1. Dataset GE7

The experimental dataset GE7 includes B-mode ultrasound images that have been acquired using a GE Logiq 7 (General Electric, USA) ultrasound machine. The parameters of the device for acquiring the ultrasound images had always the same values: Frequency of 5.5 MHz, Gain of 78, Depth of 16.0 cm, DR (Dynamic Range) of 111. Several 200 patients were analyzed in this study for dataset GE7. For each patient, the number of annotated ultrasound images varies from 3 up to 30. The patch generation procedure described in Section 2.3 is used.

### 2.2. Dataset GE9

The second experimental dataset GE9 includes B-mode ultrasound images that have been acquired using a GE Logiq 9 (General Electric, USA) ultrasound machine. The parameters of the device for acquiring the ultrasound images had always the same values: Frequency of 6 MHz, Gain of 58, Depth of 16.0 cm, DR (Dynamic Range) of 69. The ultrasound images of this dataset resulted from the ultrasound liver analysis of 68 patients. The number of annotated images per patient varies from 3 to 35 images. The patch generation procedure described in Section 2.3 is used.

### 2.3. Patch Generation Procedure

**Figure 2:**
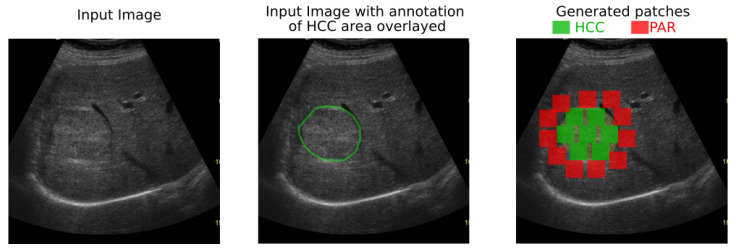
Generation of HCC and PAR patches of 56×56 pixels by scanning the marked area and its close exterior.

In both datasets, the HCC was marked as a polygonal area in the image, as shown in Figure 1—right, and Figure 2—middle. For each ultrasound image, considering the marked HCC area, rectangular image patches(regions) have been selected, by means of a controlled sliding window procedure. Part of these patches are located inside the annotated area and they correspond to the HCC class. The samples of the generated patches are depicted in green in Figure 2. Other patches are in the immediate vicinity of the polygonal annotation, but outside the marked HCC area. They correspond to the PAR samples and are shown in red in Figure 2. Cases of healthy patients were not considered because, usually, HCC evolves on cirrhotic liver tissue, for patients already affected by cirrhosis. The medical specialists suggested a focus on these two cases—to make the differentiation between HCC and the cirrhotic parenchyma on which it evolved, while healthy tissues have not been included in this study.

Due to the nature of the tumors, due to the small area they occupy in the ultrasound images and in order to ensure at least one valid HCC and one valid PAR region per annotation, a size of 56×56 pixels was selected for the regions of interest. The ultrasound image is traversed with a sliding window of size 56×56 pixels. If the window is inside the marked area, and its intersection with any other generated patch is smaller than 0.1% of their union, then the window is added to the set of HCC generated patches. If the window is outside the HCC marked area but one of its corners is on the boundary of the marked region and its intersection with any other patch is smaller than 0.1% of its area, we add it to the set of the PAR patches. To ensure variety and diversity in the selected image samples a minimal intersection factor of 0.1% was considered when generating the regions of interest.

Depending on the size of the HCC annotated area and on the number of annotated images for a patient, at least 3 HCC patches and 5 PAR patches have been generated for each patient. The selection was validated by medical specialists.

### 2.4. Ground Truth Data

To proof the efficacy of the proposed approach the two datasets GE7 and GE9 were considered. Table 1 shows the distribution of patches for each class.

**Figure 3:**
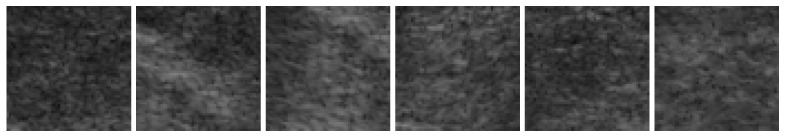
HCC patches from dataset GE7.

**Figure 4:**
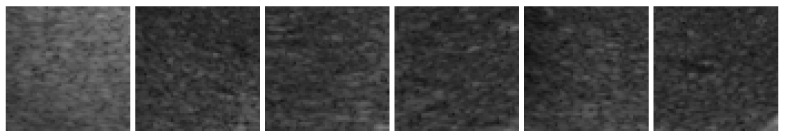
PAR patches from dataset GE7.

**Figure 5:**
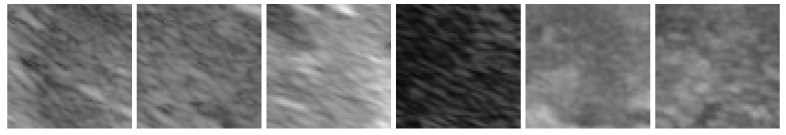
HCC patches from dataset GE9.

**Figure 6:**
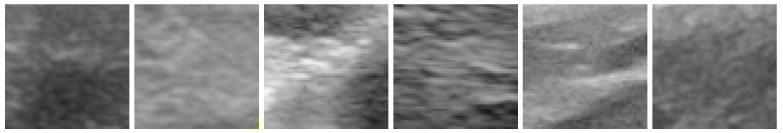
PAR patches from dataset GE9.

For training the proposed CNN model, the train dataset was augmented by means of rotation, zoom and reflection operations. Rotations in the range [−45${}^{\circ }$,45${}^{\circ }$], every 5 degrees, and zoom out/in with a factor of 0.8 and 1.2 were applied. A validation set was used for evaluating the classification performance during training. Table 2 shows the distribution of samples per class for training, for validation and for testing. Example images for each class in the two datasets are shown in Figure 3, Figure 4, Figure 5 and Figure 6.

## 3. Methods

### 3.1. Proposed Deep-Learning Based Method

The proposed solution is envisioned for a possible computer-aided diagnosis tool, which analyzes ultrasound images and offers the likelihood that a selected region of interest is of HCC type, or it represents the cirrhotic liver tissue on which it had evolved.

**Figure 7:**
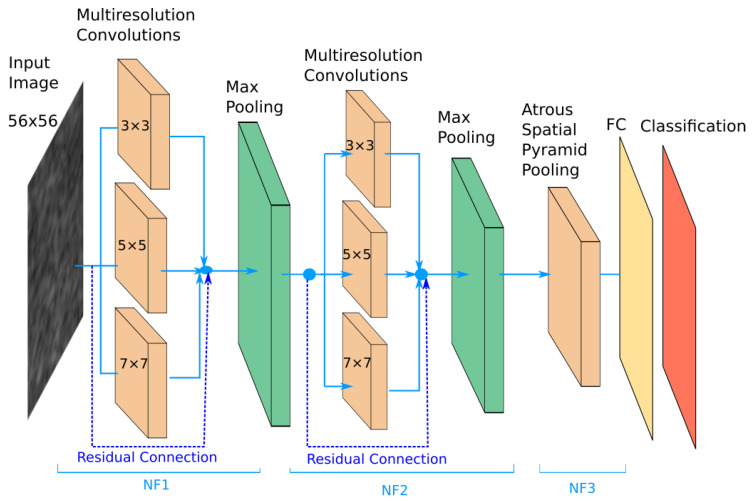
Proposed Multi-Resolution CNN.

The proposed network contains two modules of parallel multi-resolution convolutions, each followed by a down-sampling operation, an Atrous Spatial Pyramid Pooling (ASPP) module followed by a fully connected layer. The receptive field of the ASPP module is expended by means of multiple dilated convolutions which have as result a dense feature map. This expansion of the receptive field is done without loss of resolution or coverage. The proposed architecture is depicted in Figure 7.

Every convolution layer is followed by a Rectified Linear Unit (RELU) and a batch normalization(BN) layer (which for convenience of the representation are not depicted in Figure 7).

Multi-resolution features are obtained by the parallel application of convolutional filters with the kernel sizes in the set: $W=\{w_{1},w_{2},w_{3}\}$, where the size of $w_{p}$ is 2p+1 with p∈{1,2,3}. As shown in Figure 7 sizes of 3 × 3, 5 × 5 and 7 × 7 are included. Suppose we have a feature-map volume *x* which is provided as input to the multi-resolution parallel convolution block with kernels in the set *W*. The output of this block is a feature-map volume *y* obtained by the concatenation of the convolution results $y_{1}$, $y_{2}$, $y_{3}$, where:

(1) $$\begin{array}{c} y=y_{1}⨁y_{2}⨁y_{3} \end{array}$$

and the ⨁ symbol denotes the concatenation of outputs.

**Figure 8:**
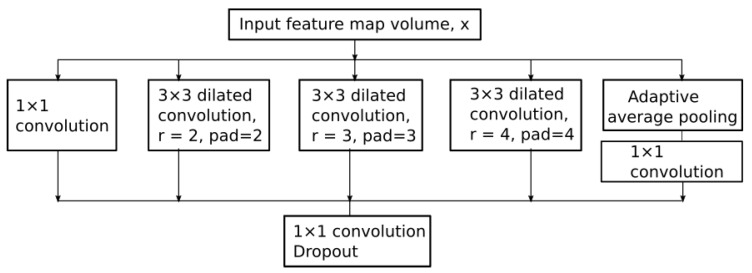
ASPP [2,39] Module Employed in the Proposed Architecture.

The Atrous Spatial Pyramid Pooling (ASPP) [39] module is located at the deepest level in the network. This module is applied on top of the feature pool extracted by the parallel multi-resolution convolutions with the role of a context module tool. The ASPP structure used in the proposed network is depicted in Figure 8.

**Figure 9:**
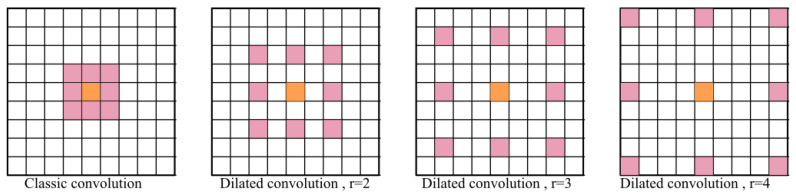
Receptive field of classical and dilated convolutions.

The five branches of the ASPP module receive an input feature-map volume *x* that represents the multi-resolution down-sampled spatial information computed by the previous layers in the network. The first branch of the ASPP module contains a 1×1 convolution that has the role of adapting the module’s input volume to its output feature-map volume. Dilated convolutions with atrous rates 2,3 and 4 are applied in parallel with an adaptive average pooling. The role of dilated convolutions is to expand the receptive fields of the feature maps. For example, if the atrous rates are 2,3,4 dilated convolutions densely sample features in the vicinity of the center pixel, as depicted in Figure 9.

The main types of layers engaged in the network topology are as follows:

- Convolutional layers that apply sliding convolutional filters with the specified stride and padding (see Figure 9).
- Dilated convolutions that perform sliding convolutional operations with the specified stride, padding and atrous sampling rate (see Figure 9)
- Batch normalization (BN) layers that have the role of normalizing the activations and gradients involved in the learning process of the neural network.
- Rectified Linear Unit layers (RELU) that perform a thresholding with respect to zero on their inputs.
- Max-pooling layers applied after each set of convolutional layers. Each pooling layer down-samples its input, and has the role of reducing the input volume and the parameter space for the subsequent layers.
- Residual connections are used for propagating features from previous layers to the next layers in the network.
- Data dropout layer is used as a regularization technique for increasing the network’s generalization capability and making it less prone to overfit the training data (see Figure 8).
- A fully connected layer combines all the features computed by the network to classify the image patches. A SoftMax function followed by a classification layer that computes the cross-entropy loss completes the model.

The proposed atrous rates (r) are tuned for the input volume that is received by the ASPP module. With the used atrous rates we accommodate the size of the filter with the size of the input feature maps. The fifth branch of the ASPP module is an adaptive average pooling used for reducing overfitting. It is followed by a 1×1 convolution that adapts its result to the output depth. The resulting concatenated feature volumes are then fed to a 1×1 convolution, followed by batch normalization and data dropout.

The number of filters for each multi-resolution layer helps controlling the size of the network’s parameter space. In the experimental part the relation between the size of the parameter space and the accuracy of the classification is analyzed throughout the results. The best configuration was obtained when NF1=NF2=NF3=128.

**Figure 10:**
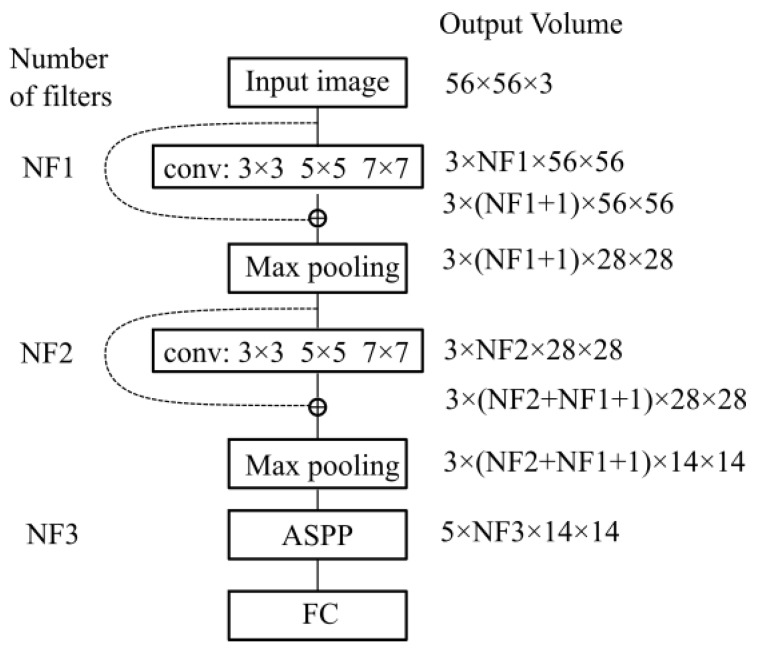
Variation in volume resolution across the network.

In Figure 10 we show the variation in volume resolution all over the blocks that constitute the proposed network configuration.

Input images of size 56×56 are forwarded to the first parallel multi-resolution convolution block that concatenates the results of the three convolutions with as output a volume of size 3 × NF1 × 56 × 56. The convolutions have a padding equal to half of the kernel size, hence the output is equal to the input resolution (56 × 56). The first shortcut connection of the network concatenates the input with the result of the first parallel convolution block, hence a volume of 3 × (NF1 + 1) × 56 × 56 results. This volume is input to the down-sampling layer which outputs a feature map of size 3 × NF2 × 28 × 28. Next, the second multi-resolution convolution block is applied. Its output is concatenated with the second shortcut connection leading to a volume of 3 × (NF2 + NF1 + 1) × 28 × 28. The second max-pooling outputs a size equal to 3 × (NF2 + NF1 + 1) × 14 × 14. This is provided to the ASPP module that contains 5 parallel branches whose output is concatenated in a volume of size 5 × NF3 × 14 × 14. This processing flow ensures a large and variate feature pool of the network.

In conclusion, the key design ideas taken into account in the proposal of the solution are:

1. Inclusion of various size convolution kernels (3 × 3, 5 × 5, 7 × 7) that ensure the extraction of different meaningful multi-resolution textural features from the input images (homogeneous areas, granular areas).
2. The consideration of an Atrous Spatial Pyramid Pooling module that samples relevant features with various densities, enriching the field of view of the multi-resolution textural features.
3. Residual connections are used to propagate the input feature maps of the current layer to its output, hence multi-resolution feature sharing throughout the network is ensured.

### 3.2. Conventional Machine-Learning (ML) Methods

To reveal the subtle properties of the hepatic tissue, various conventional texture analysis methods were taken into account and several features have been computed to be provided as input for conventional ML algorithms such as MLP, SVM, RF and AdaBoost combined with decision trees.

The Haralick features (homogeneity, energy, entropy, correlation, contrast and variance) were defined, based on the GLCM matrix, as described in [34]. These features can emphasize visual and physical properties within ultrasound images, such as heterogeneity, echogenicity, gray level disorder, gray level complexity, gray level contrast. The GLCM is defined over an image and represents the distribution of co-occurring pixel values at a given offset. The definition of the GLCM of order n is provided in (2). Thus, each element of this matrix stores the number of the n-tuples of pixels, placed at the coordinates $\left(x_{1},y_{1}\right),\left(x_{2},y_{2}\right),\ldots ,\left(x_{n},y_{n}\right)$, with the gray level values $g_{1},g_{2},\ldots ,g_{n}$, being in a spatial relationship defined by the displacement vectors, $\vec{d}$.

(2) $$\begin{array}{c} C_{D}\left(g_{1},g_{2},\ldots ,g_{n}\right)=\#\{(\left(x_{1},y_{1}\right),\left(x_{2},y_{2}\right),\ldots ,\left(x_{n},y_{n}\right):\\ I\left(x_{1},y_{1}\right)=g_{1},I\left(x_{2},y_{2}\right)=g_{2},\ldots ,I\left(x_{n},y_{n}\right)=g_{n},\\ |x_{2}-x_{1}\left|=\right|\vec{dx_{1}}\left|,\right|x_{3}-x_{1}\left|=\right|\vec{dx_{2}}\left|,\ldots ,\right|x_{n}-x_{1}\left|=\right|\vec{dx_{n-1}}|,\\ |y_{2}-y_{1}\left|=\right|\vec{dy_{1}}\left|,\right|y_{3}-y_{1}\left|=\right|\vec{dy_{2}}\left|,\ldots ,\right|y_{n}-y_{1}\left|=\right|\vec{dy_{n-1}}|,\\ sgn\left(\left(x_{2}-x_{1}\right)\left(y_{2}-y_{1}\right)\right)=sgn\left(\vec{dx_{1}}\cdot \vec{dy_{1}}\right),\ldots ,\\ sgn\left(\left(x_{n}-x_{1}\right)\left(y_{n}-y_{1}\right)\right)=sgn\left(\vec{dx_{n-1}}\cdot \vec{dy_{n-1}}\right))\} \end{array}$$

In Equation (2), # stands for the cardinal number of the set, while *I* stands for the image intensity function. The displacement vectors are provided in Equation (3):

(3) $$\vec{d}=\left(\left(\vec{dx_{1}},\vec{dy_{1}}\right),\left(\vec{dx_{2}},\vec{dy_{2}}\right),\ldots ,\left(\vec{dx_{n-1}},\vec{dy_{n-1}}\right)\right)$$

In the perfomed experiments, the second and third order GLCM were computed, (n∈{2,3}) [32]. For the second order GLCM, the absolute value of the corresponding displacement vector components was considered to be equal to 1, while the directions of these vectors varied between 0${}^{\circ }$ and 360${}^{\circ }$, being always a multiple of 45${}^{\circ }$. In the case of the third order GLCM, specific orientations of the displacement vectors were taken into account. Thus, the corresponding three pixels involved in the computation of the third order GLCM, were either collinear, or they formed a right-angle triangle, the current pixel being situated in the central position. In the case of the collinear pixels, the direction pairs were (0${}^{\circ }$,180${}^{\circ }$), (90${}^{\circ }$, 270${}^{\circ }$), (45${}^{\circ }$, 225${}^{\circ }$), (135${}^{\circ }$, 315${}^{\circ }$), while in the case of the right-angle triangle, the direction pairs were the following: (0${}^{\circ }$,90${}^{\circ }$), (90${}^{\circ }$, 180${}^{\circ }$), (180${}^{\circ }$, 270${}^{\circ }$), (0${}^{\circ }$, 270${}^{\circ }$), (45${}^{\circ }$, 135${}^{\circ }$), (135${}^{\circ }$, 225${}^{\circ }$), (225${}^{\circ }$, 315${}^{\circ }$), and (45${}^{\circ }$, 315${}^{\circ }$). The absolute values of the corresponding displacement vector components (offsets) were either 0 or 2, in this case. Finally, the Haralick features were computed, in both cases of second and third order GLCM matrices, as the arithmetic mean of the individual values, for each of the GLCM matrices, which corresponded to each combination of parameters [32]. The auto-correlation index [34] was also taken into account, as a granularity measure, while the Hurst fractal index [40] characterized the roughness of the texture. Edge-based statistics were computed as well, such as edge frequency, edge contrast and average edge orientation [32], aiming to reveal the complexity of each class of tissue. The density (arithmetic mean) and frequency of the textural micro-structures, resulted after applying the Laws’ energy transforms [40], were also included in the feature set. Multi-resolution textural features were considered to be well, such as the Shannon entropy computed after applying the Haar Wavelet transform recursively, twice. The low-low (ll), low-high (lh), high-low (hl) and high-high (hh) components were first determined on the original image, then the Haar Wavelet transform was applied again, on all these components. The Shannon entropy was determined on each component, at the first or second level, as expressed by Equation (4).

(4) $$Entropy=-\sum_{x=1}^{N}\sum_{y=1}^{M}|I\left(x,y\right)|log_{2}\left|I\left(x,y\right)\right|$$

In Equation (4), *M* and *N* are the dimensions of the region of interest, while *I* is the image intensity function [32,34]. All the textural features were computed on the rectangular regions of interest, with 56×56 pixels, after the application of the median filter (for speckle noise attenuation), independently on orientation, illumination and region of interest size.

Relevant feature selection was also performed, employing specific methods, such as Correlation-based Feature Selection (CFS), Consistency-based Feature Subset Evaluation, Information Gain Attribute Evaluation, respectively Gain Ratio Attribute Evaluation [41]. Only those features with a relevance score above the selected threshold were considered to be relevant. The final set of relevant features resulted as a union of the relevant feature sets provided by each applied method. These textural features were used, before and after feature selection, in combination with the following traditional classification methods:

- Support Vector Machines (SVM)
- Multi-layer Perceptron (MLP)
- Random Forest (RF)
- AdaBoost meta-classifier combined with the C4.5 technique for decision trees [41].

The approach presented in [42] was also taken into account for comparison. Textural features extracted from LBP were combined with GLCM features. LBP features have been introduced by [35]. To compute these features a circle of radius *R* is considered around each pixel. *N* neighboring pixels are selected from a circle of radius *R* and center of coordinates $x_{c}$, $y_{c}$. The LBP code is obtained by a sign function *s* applied to the differences between the intensity of neighbors and the intensity of the center pixel. For each neighbor if the difference is greater than 0 a code of 1 is considered otherwise a code equal to 0 is considered. The N codes form a number that represents the local binary pattern associated with that pixel.

(5) $$LBP\left(x_{c},y_{c}\right)=\sum_{p=0}^{N-1}s\left(I_{p}-I_{c}\right)\times 2^{p}$$

where $I_{p}$ is the intensity level of one of the N neighbors. Next, based on the generated codes the image is divided into non-overlapping cells and a histogram of the LBP codes is computed for each cell. The LBP histograms in combination with GLCM features were considered in the experiments, together with traditional classifiers such as SVM and AdaBoost in conjunction with decision trees.

## 4. Experimental Results

For evaluation we use the indicators extracted from the confusion matrix, as well as the AUC. We are interested in obtaining both a high sensitivity (as positive samples we consider HCC) and a high specificity (as negative samples we consider PAR regions). Training and evaluation is done on both datasets, GE7 and GE9.

### 4.1. Convolutional Neural Network (CNN) Methods

The proposed CNN model was developed in Python. During training, the parameters of the proposed multi-resolution network are set up using a uniform distribution initialized by means of the Glorot method [43]. The model is trained for 100 epochs with a mini-batch size of 64 image instances. Training convergence is achieved within 100 epochs. Stochastic Gradient Descent is adopted with a learning rate of 0.0001 and a momentum equal to 0.1. A computing framework consisting of an i7 processor, with 16 GB of memory and with a GeForce GTX 1070 GPU was used.

The influence of the number of filters in each multi-resolution convolution block and in the atrous spatial pyramid pooling block on the accuracy of the results was investigated. Several setups were examined as follows:

- Setup 1: for which NF2 = NF1/2, and NF3 = NF1/4. In this topology the first multi-resolution block has a large number of channels and while advancing through the network the feature space is decreased by the reduction in the number of filters. It can be remarked that this reduction is not efficient for the classification task.
- Setup 2: for which NF2 = NF1 × 2, NF3 = NF2 × 4. This means that the feature volume is increased throughout the network. This setup corresponds to an enlarged feature map as the network goes deeper.
- Setup 3: for which NF1 = NF2 = NF3. In this case, the number of output channels is equal between the multi-resolution blocks, but as one can notice from Figure 10 the volume is increased through the concatenation operations of the network.

The number of filters was varied during the experiments from 16, to 32, to 64, respectively to 128 filters. The best obtained results are shown in Table 3.

It can be observed that optimal results are achieved for setup 3 with NF1 = NF2 = NF3 = 128. For dataset GE7 the best result highlights an accuracy of 91% with an AUC of 95%, while for dataset GE9 we obtain an accuracy of 84.84% with an AUC of 91%.

From the experiments it can be noted that the effect of varying the number of output filters of the multi-resolution blocks has a notable effect in the performance of the network. By the analysis of setup 1 it can be noticed that a large number of filters at the first multi-resolution block for example when NF1 = 128 followed by NF2 = 64 and NF1 = 32 provides an accuracy of 86.94% for GE7 and 82.8% for GE9, which is acceptable. However, if the number of filters in the first layer is decreased for example in the situations of NF1 = 64 or NF1 = 32, and this linear decrease is applied also to NF2 and NF3 then the accuracy is diminished.

An analysis of setup 2 in which the number of filters for the first multi-resolution block is varied from 16 to 32 and to 64, while the second multi-resolution block and the ASPP have a larger number of filters, leads to a small accuracy with respect to setup 1. Hence, a larger number of filters for NF1 is beneficial for accuracy.

From our experiments it results that an equal number of output filters for each multi-resolution module and for the ASPP module, as in setup 3 with NF1 = NF2 = NF3 has the role of re-balancing the number of output channels and provides a good boost in accuracy.

For comparing the proposed solution with other CNN-based methods applied on the same datasets, five state of the art neural network architectures were considered in the process of transfer learning. They were pre-trained on the ImageNet [44] dataset, hence they all have the size of the output layer equal to 1000. They were reshaped to keep the same number of inputs but their outputs should be equal to 2. The considered architectures and the operations made to modify these networks to cope with the input datasets are:

- ResNet18 [11] (18 layers with residual connections). The last fully connected layer is reinitialized to have 512 input features and 2 output features.
- For VGGNet [8] the output of the network comes from the 6th layer of the classifier, which has 4096 input features, and its output is set to 2.
- In what regards Inceptionv3-Net [10]—it has two output layers (1) the primary output that is a linear layer at the end of the network and (2) the auxiliary output, used as a regularizer. Both the auxiliary classifier and the primary one is reshaped during the transfer learning procedure and their output parameter is set to 2.
- Densenet with 121 [12] layers is used. The output layer is a linear layer with 1024 input features. To reshape the network, we reinitialize the classifier’s linear layer output to be equal to 2.
- SqueezeNet [13] has various configurations. We have used the one provided in Pytorch, where the output of the network comes from a 1 × 1 convolutional layer, which is the 1st layer of the classifier. To reshape the network the Conv2d layer is reinitialized to have an output feature-map volume of depth 2.

**Figure 11:**
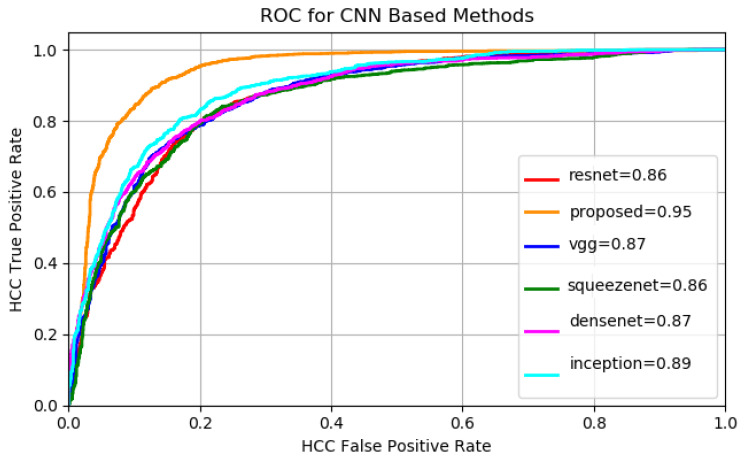
GE7 ROC for CNN Methods.

**Figure 12:**
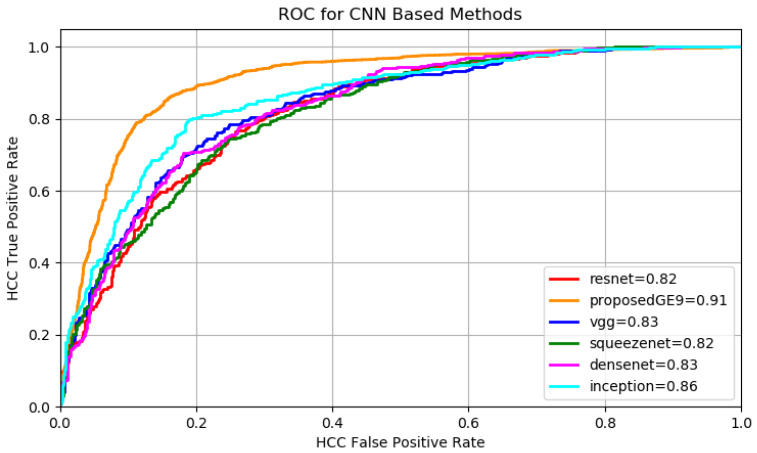
GE9 ROC for CNN Methods.

A batch size of 64 images and 100 epochs have been employed during the training of each architecture, for each of the two datasets. The variation of sensitivity with respect to the specificity was studied (see Figure 11, Figure 12 and Table 4) and the degree of separability obtained by each method using the AUC metric was considered.

In the following, the results of the proposed solution are compared with the ones obtained by transfer learning.

It can be noticed from Table 4 that the best performing fine-tuned networks, in terms of accuracy and AUC are InceptionNet [10] and Densenet [12] due to their inception-like modules and maximum information flow obtained in Densenet by multiple forward layer connections.

### 4.2. Conventional Texture-Based Classifiers

To provide a detailed comparison between the deep-learning approach and classical ML classifiers trained using textural features the following methods provided by the Weka Library [41] were included in the experiments:

- The John Platt’s Sequential Minimal Optimization (SMO), which implements SVM, the input data being normalized, the best results being achieved in the case of the polynomial kernel of 1st degree;
- The AdaBoostM1 meta-classifier, in combination with the J48 technique, the Weka equivalent of C4.5, where the number of weak learners was varied between 10 and 1000, until the best performance was achieved (in our cases for 100 weak learners).
- The RF classifier, where the value of the number of trees parameter was varied between 10 and 1000, until the best performance was achieved (in this study for 100 trees).
- The MLP classifier has been adopted, as well. By varying the number and structure of the hidden layers the best architecture for this classifier was targeted. The following topologies were taken into account for this purpose: one, two or three hidden layers, each of them with *a*, a/2 or a/3 number of nodes, where a = (number of input features + number of classes)/2. For the MLP classifier, the learning rate was 0.2, the value of α parameter was fixed to 0.8 and the training time was tuned to 500 epochs, for achieving both high speed and high accuracy of the learning process.

Several 47 textural features were determined on the considered regions of interest, using our own Visual C + + software modules, as described within Section 3.2, their values being provided at the inputs of the above mentioned traditional classifiers, before and after relevant feature selection. During the classification performance assessment, 80% of the data was included in the training set, while 20% of the data constituted the test set.

Relevant feature selection was employed in these experiments. The following methods were taken into account:

1. Correlation-based Feature Selection (CFS) in combination with genetic search, taking into account 20 generations;
2. Consistency-based Feature Subset Evaluation in combination with genetic search, considering 20 generations.
3. Information Gain Attribute Evaluation in combination with the Ranker method;
4. Gain Ratio Attribute Evaluation in combination with the Ranker method.

For the first two methods, which performed feature subset assessment, all the features belonging to the best resulted subset, with the highest merit, were included in the relevant feature set. For the last two methods, as they performed the assessment of individual attributes, only those textural features which had a significant score, above 0.3, were considered to be being relevant. The union of the relevant textural features provided by all these methods was finally taken into account.

Table 5 and Table 6 present the classification performance parameters that resulted before and after feature selection among various potentially relevant textural features and include a comparison with the deep-learning-based model.

The textural features were extracted in a similar manner with the previous approaches [32]. All these experiments were performed on a computer with an i7 Intel core processor and 8 GB of Random Access Memory (RAM). The approach presented in [42] that computes LBP and GLCM statistics, respectively trains SVM and AdaBoost classifiers on the same dataset was also considered.

In both cases, among the relevant textural features that have been selected, one can remark the homogeneity, energy, entropy, correlation, contrast, variance, derived from the second and third order GLCM matrices, the auto-correlation index, the Hurst fractal index, the Shannon entropy resulted after applying the Wavelet transform recursively, at the first level, as well as at the second level, on all the components, respectively the features resulted after the application of the Laws filters, corresponding to various types of micro-structures, such as levels, spots, waves and ripples. All these features emphasize the heterogeneous, complex, chaotic character of the HCC tissue, respectively differences in granularity between the HCC malignant tumor and the cirrhotic liver tissue on which it had evolved. It can be remarked that the classification performance increases after the feature selection process, in most of the cases. Thus, the best accuracy (recognition rate) was obtained, in both cases, after feature selection, for the SMO classifier with a polynomial kernel of first degree, the best sensitivity resulted, in the first case, for the SMO classifier, before and after feature selection, while in the second case, the best sensitivity was achieved for the MLP classifier after feature selection; the best specificity resulted, in the first case, for the MLP classifier after feature selection, while in the second case the best sensitivity resulted for both the RF classifier and the AdaBoost meta-classifier combined with the J48 method of decision trees, after feature selection; the highest AUC was obtained, in the first case for the SMO classifier that employed a polynomial kernel of first degree, before, as well as after feature selection, while in the second case, the highest AUC value resulted for the SMO classifier, after the feature selection process. In the case of the combination of LBP and GLCM, the best configuration with respect to accuracy is attained with AdaBoost classifiers.

## 5. Discussions

**Figure 13:**
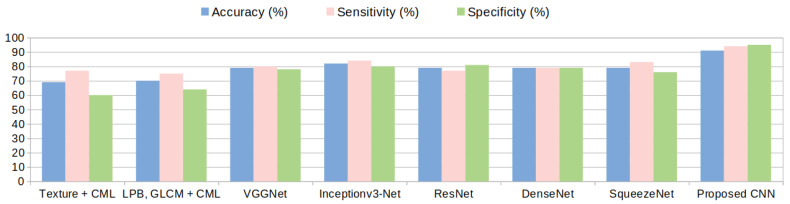
Accuracy comparison among various approaches for classifying hepatocellular carcinomavs cirrhotic parenchyma in dataset GE7.

**Figure 14:**
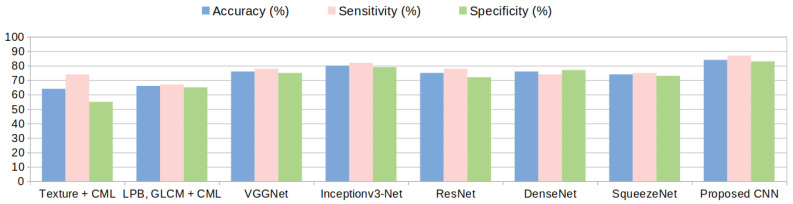
Accuracy comparison among various approaches for classifying hepatocellular carcinomavs cirrhotic parenchyma in dataset GE9.

Concerning the results, both a high sensitivity and a high specificity were targeted because for the medical specialist both are important. For each of the presented methods the cases of both high sensitivity and specificity are shown in the results Table 4, Table 5 and Table 6. It can be noticed that the proposed deep-learning-based model achieves the most relevant results (accuracy, sensitivity and AUC greater than 90%). A specificity above 88% was also obtained, which is meaningful for avoiding a false diagnosis. An accuracy comparison, taking into account all the approached methods, is depicted in Figure 13 for dataset GE7 and in Figure 14 for dataset GE9. It can be noticed again that the performance of the deep-learning methods overcame that of the traditional classification approaches and among the deep-learning architectures, the proposed deep-learning-based solution led to the best results.

Thus, it can be concluded that the performance of the newly developed deep-learning model is comparable with the state of the art results, detailed in Section 1, regarding all the parameters (accuracy, sensitivity, specificity and AUC) obtained for the best configuration.

**Figure 15:**
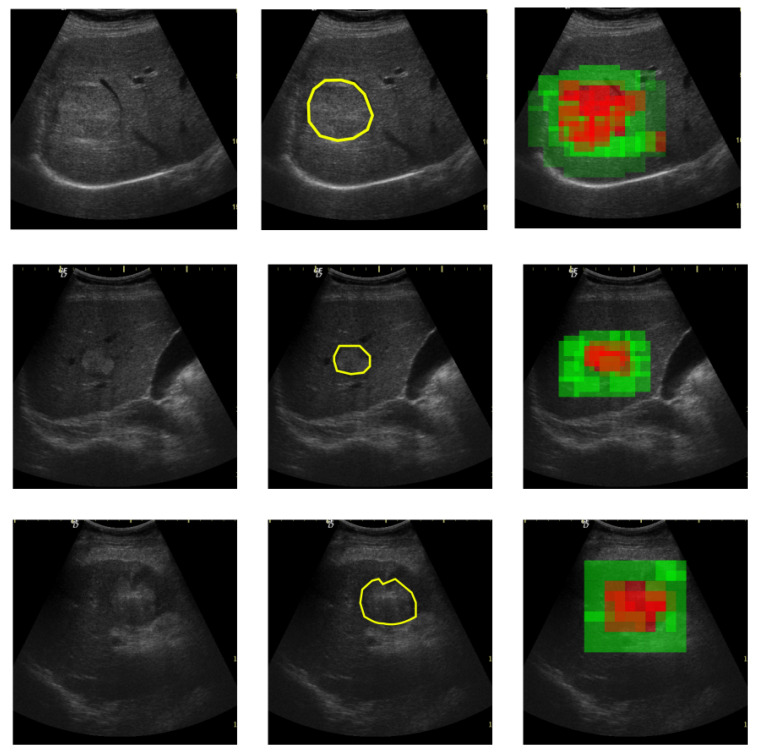
Input image (**left**), ground truth with HCC area enclosed in yellow polygon (**middle**), predicted confidence map in which HCC likelihood of a patch is marked in red and the PAR high confidence is marked in green.

On a large scale, in the context of a medical diagnosis framework, the proposed model can be used for providing a visual trigger for the medical specialist. As depicted in Figure 15, being given an input ultrasound image the medical specialist could select the interest region (marked with yellow) and the proposed method provides a confidence map for that region and its surroundings. Thus, in Figure 15 a high probability of containing a HCC patch is depicted with red and a high probability of PAR is depicted with green.

Experiments with regions that are fully contained in a large HCC area or in a large PAR area within the ultrasound image were performed. By large area it is understood that the polygonal annotation of HCC, performed by the medical specialist, has an area about 3 times larger than the size of the patches on which the model was trained (56 × 56 pixels). The model correctly identifies 94% of the HCC patches and 88% of the PAR patches, as it results from Table 3. Experiments with patches which are on the border were also performed. The border contains diffuse liver tissue that marks the transition between the HCC and PAR areas, which are very difficult to classify—that is they contain both HCC areas and PAR patches. For these situations, the model highly favors the class that has a larger area in the selection patch. As a future improvement concerning these patches, a pixel-based segmentation could be approached.

## 6. Conclusions

A deep-learning-based solution that achieves results comparable to the state of the art methods for the problem of differentiating between HCC and the cirrhotic liver tissue areas using image processing and classification techniques applied to ultrasound images was designed, implemented and experimented. The topology of the proposed deep-learning model considers the benefits of state of the art solutions for CNN-based image classification and combines their architectural particularities in a model suitable for highly textured ultrasound images of liver. The proposed CNN model combines parallel convolutions that capture multi-resolution textural features, residual connections that enable feature sharing between layers, and atrous convolutions for spatial pyramid pooling and context information generation. Extensive experiments compare the performance of the proposed method with other CNN-based image classification methods and also with conventional machine-learning techniques applied on relevant textural features extracted from ultrasound images and prove the efficiency of the proposed solution. The obtained results are highly valuable from the point of view of the medical specialist, as the final objective, in this case, is that of determining the presence or absence of the HCC tumor for patients suffering from advanced cirrhosis, hence providing a visual trigger for the medical expert that analyzes the ultrasound images. As future work we aim to enhance the experimental dataset by collecting more relevant images including benign liver tumors as well, respectively to find appropriate techniques in order to combine the images acquired using different ultrasound machines, under different settings.

## Figures and Tables

**Table 1:** Ground truth data.

Dataset	Cases	Annotated Images	HCC Patches	PAR Patches
GE7	200	823	7930	8190
GE9	68	508	5140	5200

**Table 2:** Train/test/validation set configurations.

Dataset and Class	Train	Train (Augmented)	Test	Validation
GE7 HCC	5324	53,240	1586	1020
GE7 PAR	5510	55,100	1638	1042
GE9 HCC	3312	33,120	1028	800
GE9 PAR	3360	33,600	1040	800

**Table 3:** HCC/Cirrhotic parenchyma differentiation: the performance of the proposed CNN multi-resolution method.

Dataset	Setup	Accuracy	Sensitivity	Specificity	AUC
GE7 Setup 1	NF1 = 128, NF2 = 64, and NF3 = 32	86.94%	91.75%	82.2%	93%
	NF1 = 64, NF2 = 32, and NF3 = 16	84.3%	87.2%	82%	90%
	NF1 = 32, NF2 = 16 and NF3 = 8	78.6%	80.5%	75.3%	87%
GE7 Setup 2	NF1 = 16, NF2 = 32, NF3 = 64	69.02%	64.58%	76.11%	76%
	NF1 = 32, NF2 = 64, NF3 = 128	70.09%	67.18%	73.68%	75%
	NF1 = 64, NF2 = 128, NF3 = 256	74.15%	71.6%	77.06%	79%
GE7 Setup 3	NF1 = NF2 = NF3 = 32	80.33%	84.27%	72.92%	86%
	NF1 = NF2 = NF3 = 64	88.2%	89.44%	86%	92%
	NF1 = NF2 = NF3 = 128	91%	94.37%	88.38%	95%
GE9 Setup 1	NF1 = 128, NF2 = 64, and NF3 = 32	82.8%	84.5%	81.13%	89%
	NF1 = 64, NF2 = 32, and NF3 = 16	80.10%	79.77%	80.46%	86%
	NF1 = 32, NF2 = 16 and NF3 = 8	79.74%	79.11%	80.46%	87%
GE9 Setup 2	NF1 = 16, NF2 = 32, NF3 = 64	77.66%	76.25%	79.4%	84%
	NF1 = 32, NF2 = 64, NF3 = 128	79.63%	81.16%	78.12%	85%
	NF1 = 64, NF2 = 128, NF3 = 256	82.45%	82.48%	82.42%	90%
GE9 Setup 3	NF1 = NF2 = NF3 = 32	76.79%	78.24%	72.58%	82%
	NF1 = NF2 = NF3 = 64	82.63%	81.12%	80.42%	88%
	NF1 = NF2 = NF3 = 128	84.84%	86.79%	82.95%	91%

**Table 4:** Results obtained using transfer learning.

Dataset	Method	Accuracy	Sensitivity	Specificity	AUC
Ge7	VGGNet [8]	79.46%	77.21%	78.8%	84%
	ResNet [11]	79.34%	78.66%	81.10%	85%
	InceptionNet [10]	82%	84.3%	80%	89%
	DenseNet [12]	79.46%	79.79%	79.17%	87%
	SqueezeNet [13]	79.53%	83.24%	76.77%	86%
	Proposed method	91%	94.37%	88.38%	95%
Ge9	VGGNet [8]	76.38%	78.21%	74.8%	83%
	ResNet [11]	75.26%	78.11%	72.37%	82%
	InceptionNet [10]	80.39%	81.63%	79%	86%
	DenseNet [12]	75.44%	74.24%	77.15%	83%
	SqueezeNet [13]	74.32%	75.22%	73.22%	82%
	Proposed method	84.84%	86.79%	82.95%	91%

**Table 5:** HCC/PAR differentiation for dataset GE7: the performance of the traditional classifiers before and after feature selection.

Dataset	Method	Accuracy	Sensitivity	Specificity	AUC
GE7	Before feature selection				
	SMO (poly 1)	68.5%	77%	60%	68.5%
	MLP	52.75%	57.5%	48%	55.5%
	RF	62.5%	75%	49.5%	60.9%
	AdaBoost + J48	60.75%	68%	53.5%	57.8%
GE7	After feature selection				
	SMO (poly 1)	68.75%	77%	60%	68.5%
	MLP	57%	20%	94%	46%
	RF	59%	72%	46%	60.7%
	AdaBoost + J48	63.75%	78%	49.5%	68%
GE7	No feature selection				
	AdaBoost + GLCM + LBP [42]	69.5%	75%	64%	73.5%
	SVM + GLCM + LBP [42]	64.5%	64%	65%	69%
GE7	Proposed CNN	91%	94.37%	88.38%	95%

**Table 6:** HCC/PAR differentiation for dataset GE9: the performance of the traditional classifiers before and after feature selection.

Dataset	Method	Accuracy	Sensitivity	Specificity	AUC
GE9	Before feature selection				
	SMO (poly 1)	62.5%	72.2%	52.8%	62.5%
	MLP	58.2%	76%	40.4%	59.8%
	RF	58.55%	62.7%	54.4%	63%
	AdaBoost + J48	55.2%	49.8%	60.6%	60.3%
GE9	After feature selection				
	SMO (poly 1)	63.86%	73.1%	54.7%	63.9%
	MLP	58.99%	70.7%	47.4%	61.5%
	RF	56.93%	52.4%	61.5%	61.7%
	AdaBoost + J48	56.93%	52.4%	61.5%	61.7%
GE9	No feature selection				
	AdaBoost + GLCM + LBP [42]	66%	67%	65.7%	72%
	SVM + GLCM + LBP [42]	65%	61.5%	69%	71%
GE9	Proposed CNN	84.84%	86.79%	82.95%	91%

## Abbreviations

The following abbreviations are used in this manuscript:

ANN	Artificial Neural Networks
ASPP	Atrous Spatial Pyramid Pooling
AUC	Area Under the Curve
BN	Batch Normalization
CFS	Correlation-based Feature Selection
CNN	Convolutional Neural Network(s)
conv.	convolution
DICOM	Digital Imaging and Communications in Medicine
GLCM	Gray Level Co-occurrence Matrix
HCC	Hepatocellular Carcinoma
LBP	Local Binary Patterns
ML	Machine Learning
MLP	Multi-layer Perceptron
MP	Max Pooling
PAR	cirrhotic parenchyma
RF	Random Forest
SMO	Sequential Minimal Optimization
SVM	Support Vector Machines
